# 782 Achieving Optimal Care in Burns Using Enhanced Recovery After Surgery Guidelines

**DOI:** 10.1093/jbcr/irac012.333

**Published:** 2022-03-23

**Authors:** Sanja Sljivic, Lori Chrisco, Rabia Nizamani, Booker King, Felicia Williams

**Affiliations:** University of North Carolina - Chapel Hill, Chapel Hill, North Carolina; North Carolina Jaycee Burn Center, Chapel Hill, North Carolina; UNC Jaycee Burn Center, Chapel Hill, North Carolina; UNC Jaycee Burn Center, Chapel Hill, North Carolina; UNC Jaycee Burn Center, Chapel Hill, North Carolina

## Abstract

**Introduction:**

Burns are a major cause of accidental injury and death in the United States. Based on reports from the Centers for Disease Control and Prevention (CDC), approximately 1.1 million burn injuries require medical care each year. Even though 96.7% of individuals treated at burn centers will survive, many of the more extensive injuries require multiple surgical procedures, extensive pain control regimens, and prolonged intubation and hospitalization. We aim to establish a set of guidelines addressing the pre-, intra- and post-operative care of our burn patients.

**Methods:**

Over the years, there has been a profound interest in developing and implementing an Enhanced Recovery after Surgery (ERAS) protocol, such as those already in place across a range of surgical subspecialties. Since its initial development in 1997 by Henrik Kehlet for colorectal surgery, ERAS has evolved into a multidisciplinary approach involving surgeons, anesthesiologists, pharmacists, nutritionists, and nursing staff. It is an evidence-based multimodal protocol focused on lowering recovery time and post-operative complications while also addressing the entire patient journey from admission to discharge. An ERAS protocol for Burn Surgery has yet to be created and will need to focus on some of the major challenges involved in the care of burn patients including fluid management, pain control, nutritional status, potential prolonged ventilation, and long-term rehabilitation.

**Results:**

This set of guidelines will address the pre-operative care of our burn patients (e.g., acetaminophen and pregabalin/gabapentin on-call to the operating room), as well as the intra- and post-operative care (e.g., periodic lactate levels, operating room temperature at 85 degrees Fahrenheit, continued ketamine infusion for burns > 30% TBSA). These guidelines will be further evaluated in a clinical setting via a feasibility study to determine whether they would improve the overall outcome of our burn patients.

**Conclusions:**

An ERAS protocol in Burn Surgery needs to address the challenges and complexities of treating burn patients, and should be aimed at the pre-, intra-, and post-operative care of this patient population.

